# Complete Genome Sequences of Six Salmonella enterica Strains (S. enterica subsp. *arizonae*, S. enterica subsp. *diarizonae*, and S. enterica subsp. *salamae*) Isolated from Poultry Houses

**DOI:** 10.1128/MRA.00768-21

**Published:** 2021-10-14

**Authors:** Alexandre Lamas, Patricia Regal, Alberto Cepeda, Carlos Manuel Franco

**Affiliations:** a Food Hygiene, Inspection, and Control Laboratory, Department of Analytical Chemistry, Nutrition, and Bromatology, Universidade de Santiago de Compostela, Lugo, Spain; University of Maryland School of Medicine

## Abstract

Non-*enterica* subspecies of Salmonella enterica are associated with cold-blood animals. We report the complete genomes of six S. enterica strains (one S. enterica subsp. *arizonae* strain, four S. enterica subsp. *salamae* strains, and one S. enterica subsp. *diarizonae* strain) isolated from Spanish poultry houses. This will increase our knowledge of these non-*enterica* subspecies.

## ANNOUNCEMENT

Here, we report the complete genomes of four strains of Salmonella enterica subsp. *salamae*, one strain of S. enterica subsp. *arizonae*, and one strain of S. enterica subsp. *diarizonae,* all of which were isolated from poultry houses between 2012 and 2015. Strains were isolated from environmental samples from Spanish poultry farms following standard ISO 6579:2002/AMD 1:2007 (1). Briefly, boot swabs or sponges were incubated with 225 ml of buffered peptone water (Merck Millipore, Germany) for 18 h at 37°C. Then, 100 μl was transferred to modified Rappaport-Vassiliadis semisolid agar (Becton Dickinson, Le Pont de Claix, France) and incubated for 48 h at 41.5°C. Suspect samples were spread on XLD agar (Oxoid, Hampshire, UK) and SM-ID2 medium (bioMérieux, Marcy-l'Étoile, France) and incubated for 24 h at 37°C. Typical colonies were purified on nutrient agar (PanReac, Barcelona, Spain). API 20E (bioMérieux) and Salmonella Microlatex (Microgen Bioproducts, Camberley, UK) kits were used to confirm the Salmonella genus. Isolated strains were serotyped according to the Kauffman-White-Le Minor scheme by using standard antisera (Bio-Rad, Marnes-la-Coquette, France). Samples were stored in tryptic soy broth (TSB) (Oxoid) with 20% glycerol at –20°C until further use.

For DNA isolation, strains were growth in TSB (Oxoid) for 18 h at 37°C. One milliliter of TSB was collected and centrifuged at 16,000 × *g* for 10 min. The supernatant was discarded, and DNA was isolated from the pellet using the PureLink genomic DNA minikit (Invitrogen, Thermo Fisher Scientific, Carlsbad, CA) according to the manufacturer’s protocol. DNA was prepared with the rapid barcoding kit (SQK-RBK004; Oxford Nanopore Technologies [ONT], Oxford, UK), loaded into a R9.4.1 FLO-MIN106 flow cell (FAO81877; ONT), and sequenced using the MinION system (ONT). Raw data were collected with MinKNOW v4.2.8 software (ONT) and saved in Fast5 files. Guppy v5.0.11 (ONT) in high-accuracy mode was used for base calling, demultiplexing, and barcode trimming. Generated Fastq files were uploaded to the Galaxy Europe platform (https://usegalaxy.eu) (2), and Unicycler v0.4.8 (3) was used to create the assembly. Thus, reads were assembled with Miniasm (4) and polished with Racon v1.4.7 (5). Makeblastdb v2.9.0 and TBLASTN v2.9.0 tools (6) were used to rotate the genome starting with the *dnaA* or *rpaA* gene in the forward strand. Finally, a second polishing round was carried out with Medaka v1.3.2 (https://github.com/nanoporetech/medaka) (ONT) to obtain the final consensus sequence. One final contig was obtained for each strain.

The final assembly was annotated with the NCBI Prokaryotic Genome Annotation Pipeline (PGAP) v5.2 (7). Then, SISTR v1.1.1 (8) was used to compare the *in silico* serotyping with conventional antiserum serotyping. Antimicrobial resistance genes were searched with ResFinder v4.1 (9) (https://cge.cbs.dtu.dk/services/ResFinder). Salmonella pathogenicity islands (SPIs) were determined with SPIFinder 2.0 (10) (https://cge.cbs.dtu.dk/services/SPIFinder), modifying the parameter for threshold minimum identity to 90%. The presence of phages in bacterial assemblies was determined by using the web server PHASTER in July 2021 (https://phaster.ca) (11). Default parameters were used except where indicated. Results of these analyses are shown in Table 1. The genomes obtained in this study will be useful to gain a deeper understanding of the pathogenic potential of these non-*enterica* subspecies.

**TABLE 1:** Genomic characteristics of S. enterica subsp. *diarizonae*, S. enterica subsp. *arizonae*, and S. enterica subsp. *salamae* strains isolated from Spanish poultry farms

Strain	Serotype	Predicted serovar^*a*^	Length sequenced (Mb)	No. of reads	Read *N*_50_ (bp)	Genome size (bp)	GC content (%)	No. of coding sequences	GenBank accession no.	Raw read SRA accession no.	Resistance gene	SPIs	No. of intact prophages
LHICA D1	Salmonella enterica subsp. *diarizonae* 48:i:z	O antigen ND	638.4	137,138	9,923	4,898,223	51.7	4,577	CP078142.1	SRX9861655 to SRX9861690	*aac(6′)-Iaa*	SPI-1, SPI-2, SPI-3, SPI-5, SPI-9, SPI-13, C63PI	4
LHICA AZ23	Salmonella enterica subsp. *arizonae* 48:z4,z23:−	IIIa 48:z4,z23:−	2,809	430,871	14,015	4,402,954	51.5	4,075	CP079713.1	SRX11448516 to SRX11448627		SPI-1, SPI-2, SPI-3, SPI-5, SPI-9, SPI-13, SPI-14, C63PI	1
LHICA E3	Salmonella enterica subsp. *salamae* 4,12:b:−	II 1,4,12,[27]:b:[e,n,x]	3,588	379,520	18,605	5,081,513	51.8	4,816	CP079839.1	SRX9861803 to SRX9861904	*aac(6′)-Iaa*	SPI-1, SPI-2, SPI-3, SPI-4, SPI-9, SESS-LEE	4
LHICA SA1	Salmonella enterica subsp. *salamae* 4,12:b:−	II 1,4,12,[27]:b:[e,n,x]	506.2	65,946	16,689	5,045,976	51.8	4,758	CP079836.1	SRX9861426 to SRX9861443	*aac(6′)-Iaa*	SPI-1, SPI-2, SPI-3, SPI-4, SPI-9, SESS-LEE	3
LHICA SA2	Salmonella enterica subsp. *salamae* 6,8:g,m,t:−	H1 antigen ND	748.7	102,111	14,527	4,786,296	51.9	4,452	CP079837.1	SRX9861444 to SRX9861471	*aac(6′)-Iaa*	SPI-1, SPI-2, SPI-3, SPI-5, SPI-9, C63PI	1
LHICA SA3	Salmonella enterica subsp. *salamae* 4,12:b:−	II 1,4,12,[27]:b:[e,n,x]	3,123	430,692	13,706	4,970,704	51.8	4,640	CP079838.1	SRX9862217 to SRX9862329	*aac(6′)-Iaa*	SPI-1, SPI-2, SPI-3, SPI-4, SPI-9, SESS-LEE	2

a ND, not determined.

### Data availability.

Raw reads and assembled genomes were deposited in GenBank under BioProject accession number PRJNA681856. The GenBank accession numbers for the individual strains are CP078142.1 (LHICA D1), CP079713.1 (LHICA AZ23), CP079839.1 (LHICA E3), CP079836.1 (LHICA SA1), CP079837.1 (LHICA SA2), and CP079838.1 (LHICA SA3).
